# Neural Correlates of Autobiographical Memory: Evidence From a Positron Emission Tomography Study in Patients With Mild Cognitive Impairment and Alzheimer's Disease

**DOI:** 10.3389/fpsyt.2021.730713

**Published:** 2021-09-13

**Authors:** Claudia Frankenberg, Johannes Pantel, Uwe Haberkorn, Christina Degen, Monte S. Buchsbaum, Christina J. Herold, Johannes Schröder

**Affiliations:** ^1^Section of Geriatric Psychiatry, University Hospital Heidelberg, Heidelberg, Germany; ^2^Institute of Gerontology, University of Heidelberg, Heidelberg, Germany; ^3^Institute of General Practice, Goethe University Frankfurt, Frankfurt, Germany; ^4^Department of Nuclear Medicine, University Hospital Heidelberg, Heidelberg, Germany; ^5^Department of Psychiatry, University of California, San Diego, San Diego, CA, United States

**Keywords:** positron-emissions-tomography, Alzheimer's disease, mild cognitive impairment, neural correlates, autobiographical memories

## Abstract

**Background:** Autobiographical memory (AM) changes are the hallmark of Alzheimer's disease (AD) and mild cognitive impairment (MCI). In recent neuroimaging studies, AM changes have been associated with numerous cerebral sites, such as the frontal cortices, the mesial temporal lobe, or the posterior cingulum. Regional glucose uptake in these sites was investigated for underlying subdimensions using factor analysis. Subsequently, the factors were examined with respect to AM performance in a subgroup of patients.

**Methods:** Data from 109 memory clinic referrals, who presented with MCI (*n* = 60), mild AD (*n* = 49), or were cognitively intact, were analyzed. The glucose metabolic rates determined by positron emission tomography (PET) with ^18^F-fluorodeoxyglucose (FDG) in 34 cerebral sites important for AM were investigated for underlying subdimensions by calculating factor analysis with varimax rotation. Subsequently, the respective factor scores were correlated with the episodic and semantic AM performance of 22 patients, which was measured with a semi-structured interview assessing episodic memories (characterized by event-related emotional, sensory, contextual, and spatial–temporal details) and personal semantic knowledge from three periods of life (primary school, early adulthood, and recent years).

**Results:** Factor analysis identified seven factors explaining 69% of the variance. While patients with MCI and AD showed lower values than controls on the factors *frontal cortex, mesial temporal substructures*, and *occipital cortex*, patients with MCI presented with increased values on the factors *posterior cingulum* and *left temporo-prefrontal areas*. The factors *anterior cingulum* and *right temporal cortex* showed only minor, non-significant group differences. Solely, the factor *mesial temporal substructures* was significantly correlated with both episodic memories (*r* = 0.424, *p* < 0.05) and personal semantic knowledge (*r* = 0.547, *p* < 0.01) in patients with MCI/AD.

**Conclusions:** The factor structure identified corresponds by large to the morphological and functional interrelations of the respective sites. While reduced glucose uptake on the factors frontal cortex, mesial temporal substructures, and occipital cortex in the patient group may correspond to neurodegenerative changes, increased values on the factors posterior cingulum and left temporo-prefrontal areas in MCI may result from compensatory efforts. Interestingly, changes of the mesial temporal substructures were correlated with both semantic and episodic AM. Our findings suggest that AM deficits do not only reflect neurodegenerative changes but also refer to compensatory mechanisms as they involve both quantitative losses of specific memories and qualitative changes with a semantization of memories.

## Introduction

Autobiographical memory (AM) refers to a form of declarative, long-term memory comprising knowledge of a person's own past. Changes in AM are typically observed in healthy aging, e.g., by a reduction of specificity and temporo-spatial or perceptual details (1). However, pronounced losses of AM, including important events of one's own past, form one of the clinical hallmarks of Alzheimer's disease (AD) (2) and may affect central components of the subjective sense of identity (3), which illustrates the particular importance of AM. These deficits can already be demonstrated in mild cognitive impairment (MCI), which is generally considered as the potential preclinical state of the condition and progressively makes AM inaccessible (4).

As part of declarative memory, AM comprises semantic facts and episodic events. Semantic AM contains general facts from different periods of lifetime, such as names or addresses, while episodic AM includes singular events that can be recalled with a richness of details and a feeling of re-experience when recalled (5, 6).

Neuroimaging studies identified numerous cerebral sites, including the frontal, temporal, and posterior regions, to be involved in declarative AM. As reviewed by Svoboda et al. (7) in their seminal work, this heterogeneity of brain regions may refer to a network including the medial and ventrolateral prefrontal cortices, medial and lateral temporal cortices, temporoparietal junction, retrosplenial/posterior cortices, and cerebellar structures. This heterogeneity of cerebral sites may support the different aspects of AM such as encoding/retrieval processes, emotional connotation, or semantic vs. episodic aspects. However, the 26 superordinate brain regions reviewed were derived from 24 positron emission tomography (PET) or functional MRI (fMRI) studies, but not examined in a single study (7).

In the present study, we sought to investigate these cerebral sites important for AM described by Svoboda et al. (7) for underlying subdimensions or cerebral networks. Following previous studies by our group (8, 9), this was done by calculating a factor analysis of the glucose metabolic rates obtained by PET in 129 subjects from a memory clinic. The factors identified were contrasted between patients with MCI and AD and the cognitively intact controls (cognitively healthy, HC) and subsequently correlated with clinical AM performance.

## Methods

### Participants

The data of 109 patients with MCI (*n* = 60) or mild AD (*n* = 49; NINCDS-ADRDA criteria) and HC controls (*n* = 20) who had undergone PET with ^18^F-fluorodeoxyglucose (FDG) as part of the routine diagnostic workup in the memory clinic of the Section of Geriatric Psychiatry at Heidelberg University, Germany, were included. As described in previous studies by our group (8, 10, 11), MCI was diagnosed according to the criteria of Aging-Associated Cognitive Decline (12), which consider both subjective impairment as reported by the patient or a reliable informant and deficits in a broad spectrum of cognitive domains. Deficits in relevant cognitive domains were indicated by a neuropsychological test performance of at least one standard deviation below normal age and educational levels regarding the following: memory and learning, attention and concentration, abstract thinking (problem solving and abstraction), language, and visuospatial functioning. The clinical evaluation comprised the ascertainment of personal medical and psychological health through a detailed physical and psychiatric examination. Particular care was taken to exclude subjects with coexisting severe psychiatric or medical conditions. Along with this, secondary causes of dementia were excluded by an additional screening with structural neuroimaging methods such as MRI or by analyzing biomarkers such as tau protein in the cerebrospinal fluid. The study was approved by the local ethical committee. After complete description of the study to the subjects, oral and written informed consent was obtained.

### Neuropsychological Examination

As described in previous studies by our group (10, 13), cognitive deficits were assessed using the CERAD-NP (Consortium to Establish a Registry for Alzheimer's Disease—Neuropsychological Test Battery/German version) (14), the logical memory subtests (immediate recall and delayed recall) from the Wechsler Memory Scale (WMS-R) (15), and the Trail Making Test (TMT) (16, 17).

### Examination of Autobiographical Memory

Autobiographical memory was investigated using a semi-structured autobiographical interview (Erweitertes Autobiographisches Gedächtnis Inventar, E-AGI) (18) based on the ABM Interview of Kopelman et al. (19) and the Autobiographical Interview of Levine et al. (20). The E-AGI considers both personal semantic facts (SAM; maximum score: 5) and free recalled autobiographical events (EAM), which were scored according to the number of the remembered details (maximum score: 11). To integrate the E-AGI into the routine diagnostic testing, the interview was abbreviated to three of the original five different lifetime periods: primary school, early adulthood, and recent 5 years. Further methodological details, including the psychometric properties of the E-AGI, have been described in previous studies (1, 4).

### Positron Emission Tomography

The cerebral metabolic rates (CMR) were determined using PET with FDG as a tracer. All the normalized images were smoothed with 8-mm filter prior to the Statistical Parametric Mapping (SPM) analysis. Anatomical magnetic resonance (MR) images were resectioned to the standard Talairach–Tournoux position using the protocol of Woods et al. (22) and a six-parameter rigid body transformation. ^18^F-fluorodeoxyglucose images were spatially normalized to their own respective templates with the SPM software, then co-registered to each individual's anatomical MR image with the six-parameter transformation and the FMRIB Linear Image Registration Tool (23), as described in Lehrer et al. (24). ^18^F-fluorodeoxyglucose images were normalized by dividing each voxel by the mean values of the whole brain, masked with MNI152_T1_2mm.nii brain and using slices above the Montreal Neurological Institute (MNI) *z* = −53. A restricted vertical range was chosen to minimize small errors in the brain extraction routine at low slice levels. These relative FDG metabolic rates were used in all analyses. For analyses of the Brodmann areas, the gyri, hippocampus, insula, and subcortical structures, the FDG uptake values were obtained using AFNI regions of interest (25). Details on the quality and reliability of the co-registration procedure are described in another dataset in Vyas et al. (26).

### Data Analysis

In a first step, the CMR obtained in the cerebral sites identified by Svoboda et al. (7) to be involved in AM were investigated for underlying subdimensions by calculating a factor analysis with varimax rotation, retaining the brain regions as displayed in **Table 2** sorted according to their factor loadings in a descending order. The number of factors retained was determined after analyses of the scree plot. Subsequently, the scores for each of the factors were calculated for each individual subject by averaging the items weighing |0.50| or above on each factor. This was done using the factor structure of the patients with AD and MCI, as we expected the greater variability in the mesial temporal substructures in patients with AD or MCI to result in a better definition of the structure of the factors. Subsequently, the factor scale values of the patients with MCI/AD and the healthy controls were compared with a univariate analysis of variance (ANOVA) (see **Table 3**).

The respective factor scores, which represent patterns of cerebral activity, were correlated with the episodic and semantic AM performance, which was gathered in 22 patients with MCI (*n* = 9) or mild AD (*n* = 13). Therefore, Pearson's correlation coefficients were computed based on the mean results of the episodic and semantic AM performance.

The demographic and clinical data were compared between the diagnostic groups by calculating ANOVAs with *post-hoc* Duncan's test or χ^2^-tests, where appropriate. The raw scores of the clinical data were *z*-transformed according to age-, education-, and gender-specific norms. All computations were calculated using IBM SPSS Statistics (version 25; Chicago, IL, USA); the level of significance was set at 0.05 (see Table 1).

**Table 1:** Master sample: demographical and clinical description.

	**HC**	**MCI**	**AD**	**χ^**2**^/*F*_**(df)**_**	**Group comparisons**
	**(*n* = 20)**	**(*n* = 60)**	**(*n* = 49)**		
Sex (M/F)	12/8	34/26	26/23	χ${}_{\left(2\right)}^{2}$ = 0.31	
Age (years)	68.30 ± 8.14	69.48 ± 7.75	73.06 ± 6.87	*F*_(2, 126)_ = 4.25^a^	AD > MCI
Education (years)	14.90 ± 3.35	13.85 ± 3.83	13.81 ± 3.69	*F*_(2, 126)_ = 1.34	
MMSE	28.70 ± 0.97	26.95 ± 1.76	23.06 ± 1.68	*F*_(2, 126)_ = 115.30^***^	HC > MCI and AD; MCI > AD
VF	−0.22 ± 1.02	−0.42 ± 1.13	−1.41 ± 0.92	*F*_(2, 120)_ = 14.68^***^	HC and MCI > AD
BT	0.44 ± 0.64	−0.35 ± 1.89	−2.63 ± 2.62	*F*_(2, 125)_ = 14.58^***^	HC and MCI >AD
WDL-I	−0.01 ± 1.01	−1.41 ± 1.29	−2.61 ± 1.16	*F*_(2, 124)_ = 34.65^***^	HC > MCI and AD; MCI > AD
WDL-D	−0.12 ± 1.12	−1.17 ± 1.60	−2.40 ± 1.83	*F*_(2, 124)_ = 15.71^***^	HC > MCI and AD; MCI > AD
WDL-R	−0.23 ± 1.33	−1.55 ± 2.63	−2.90 ± 3.10	*F*_(2, 124)_ = 8.75^***^	HC > MCI and AD; MCI > AD
CP	0.38 ± 1.38	−1.55 ± 2.63	−2.90 ± 3.10	*F*_(2, 124)_ = 12.90^a^	HC and MCI > AD
CP-D	0.32 ± 0.80	−0.95 ± 1.62	−2.27 ± 1.99	*F*_(2, 124)_ = 18.69^***^	HC > MCI and AD; MCI > AD
TMT-A	−0.60 ± 1.69	−1.69 ± 3.49	−3.43 ± 4.09	*F*_(2, 124)_ = 5.52^**^	HC and MCI > AD
TMT-B	−0.59 ± 2.33	−2.68 ± 3.90	−5.19 ± 4.05	*F*_(2, 117)_ = 11.32^***^	HC > MCI and AD; MCI > AD
LM-I	−0.05 ± 0.61	−1.72 ± 1.29	−2.63 ± 1.19	*F*_(2, 124)_ = 0.35	
LM-D	−0.17 ± 0.79	−1.87 ± 1.35	−2.92 ± 0.96	*F*_(2, 124)_ = 0.35	
EAM		6.56 ± 3.68	6.92 ± 2.64	*F*_(1, 21)_ = 0.08	
SAM		4.30 ± 0.74	4.18 ± 0.89	*F*_(1, 21)_ = 0.11	

*AD, Alzheimer's dementia; BT, Boston Naming Test; CP, constructive praxis; CP-D, constructive praxis delayed; df, degrees of freedom; EAM, episodic autobiographical memory; SAM, semantic autobiographical memory; HC, cognitively healthy; MCI, mild cognitive impairment; MMSE, Mini-Mental State Examination (21); TMT-A, Trail Making Test A; TMT-B, Trail Making Test B; LM-D, logical memory delayed recall; LM-I, logical memory immediate recall; WDL-D, word list delayed recall; WDL-I, word list immediate recall; WDL-R, word list recognition.*

** *p ≤ 0.01;*

*** *p ≤ 0.001*.

a *Alpha set at 0.05*.

## Results

### Demographical and Clinical Characteristics

The demographic and clinical characteristics of the master sample are summarized in Table 1. For the diagnostic groups, no significant differences emerged between sex and years of education, while those in the AD group were significantly older than those in the MCI group. Regarding the neuropsychological tests, with the exception of the logical memory tests (immediate and delayed recall), the HC group and the MCI group revealed a significantly higher performance in all tests (HC/MCI > AD). In comparison to HC, patients with MCI showed a significantly lower performance in the memory tasks (immediate and delayed recall of a word list and their recognition and the constructional praxis recall), executive functioning (Trail Making Test B), and Mini-Mental State Examination (MMSE) (see Table 1 for further details).

### Factor Analysis and Correlations With AM

Factor analysis identified seven factors (see Table 2) explaining 69% of the variance [namely, “frontal cortex” (14.55%), “mesial temporal substructures” (13.43%), “posterior cingulum” (9.70%), “occipital cortex” (9.35%), “left temporo-prefrontal areas” (8.01%), “anterior cingulum” (7.34), and “right temporal cortex” (7.15%)]. A comparison of the groups is displayed in Table 3. Relative to healthy controls, patients with AD or MCI showed significantly lower values on the factors frontal cortex, mesial temporal substructures, and occipital cortex. Patients with MCI had significantly higher values on the factors posterior cingulum and left temporo-prefrontal areas than did the controls or the controls and AD patients, respectively. The factors anterior cingulum and right temporal cortex showed only minor, non-significant group differences. Regarding associations with AM, solely, the factor mesial temporal substructures was significantly correlated with both episodic memories (*r* = 0.424, *p* < 0.05) and personal semantic knowledge (*r* = 0.547, *p* < 0.01) in patients with MCI/AD (further correlations are available from the authors on request).

**Table 2:** Factor loadings for the cerebral regions considered after varimax rotation.

	**Factor 1**	**Factor 2**	**Factor 3**	**Factor 4**	**Factor 5**	**Factor 6**	**Factor 7**
BA 45, L	0.851	−0.013	−0.084	−0.082	−0.041	0.166	−0.025
BA 9, R	0.819	−0.120	−0.068	0.169	−0.158	−0.296	0.071
BA 46, L	0.798	−0.045	0.019	−0.181	0.071	−0.072	−0.147
BA 45, R	0.795	0.071	−0.082	−0.080	−0.133	0.130	0.132
BA 9, L	0.787	−0.110	0.002	0.214	0.040	−0.242	−0.217
BA 46, R	0.779	−0.066	−0.019	−0.110	−0.051	−0.056	0.104
BA 44, R	0.722	−0.034	−0.046	−0.080	−0.116	0.181	0.194
BA 10, R	0.707	−0.223	0.109	0.118	−0.026	−0.182	0.152
BA 47, L	0.704	0.311	−0.109	−0.009	−0.145	0.257	−0.118
BA 44, L	0.702	−0.126	−0.066	0.009	0.104	0.253	0.031
BA 10, L	0.698	−0.254	0.064	−0.036	0.208	−0.087	−0.079
BA 47, R	0.672	0.331	−0.078	0.084	−0.301	0.173	0.096
BA 8, R	0.670	−0.134	−0.146	0.172	−0.097	−0.497	−0.056
BA 8, L	0.606	−0.137	−0.026	0.038	0.102	−0.481	−0.278
Hippocampus, L	−0.557	0.466	0.103	0.384	0.111	0.073	−0.258
Hippocampus, R	−0.436	0.399	0.204	0.365	−0.251	0.056	0.256
BA 28, L	−0.040	0.881	−0.015	0.123	0.192	0.083	0.009
BA 34, L	−0.099	0.808	−0.082	0.315	−0.124	−0.038	−0.200
BA 28, R	−0.036	0.793	0.014	0.107	−0.087	0.041	0.461
BA 34, R	−0.188	0.790	−0.006	0.299	−0.230	−0.041	0.032
Amygdala, L	−0.138	0.774	0.037	0.013	0.248	0.241	0.001
BA 35, R	−0.113	0.770	−0.054	0.051	−0.131	0.073	0.368
BA 35, L	−0.046	0.744	−0.054	−0.195	0.305	0.214	0.066
Amygdala, R	−0.179	0.671	0.177	0.053	−0.110	0.228	0.475
BA 25, L	0.177	0.637	0.016	0.288	−0.257	0.114	−0.267
BA 25, R	0.165	0.598	0.057	0.330	−0.349	0.079	−0.031
BA 36, L	−0.368	0.596	0.109	−0.066	0.541	0.162	0.110
BA 38, L	0.024	0.583	−0.164	0.267	0.332	0.236	0.074
BA 27, R	−0.202	0.473	0.100	0.441	−0.171	−0.034	0.033
BA 27, L	−0.352	0.421	0.117	0.399	0.122	0.010	−0.329
BA 23, R	0.066	−0.019	0.892	0.085	−0.087	0.051	0.062
BA 23, L	−0.017	−0.008	0.883	0.182	0.073	0.115	−0.078
BA 31, L	0.010	−0.047	0.796	0.162	0.079	−0.136	−0.269
BA 29, L	−0.258	0.029	0.754	−0.057	0.072	0.060	−0.016
BA 29, R	−0.096	−0.040	0.750	−0.050	−0.164	0.014	0.210
BA 31, R	0.185	−0.030	0.718	0.008	−0.213	−0.179	0.044
BA 30, R	−0.098	−0.090	0.712	−0.349	−0.014	0.158	0.074
BA 30, L	−0.288	−0.025	0.674	−0.161	0.314	0.186	−0.113
BA 6, L	0.362	−0.281	−0.421	−0.420	0.084	0.016	−0.190
BA 18, L	0.009	−0.078	0.002	−0.822	0.196	0.023	−0.156
BA 18, R	0.070	−0.209	−0.014	−0.776	−0.137	−0.099	0.064
BA 17, L	−0.092	−0.033	0.022	−0.717	0.243	0.031	−0.108
BA 17, R	0.000	−0.138	−0.036	−0.683	−0.103	−0.087	−0.161
BA 19, R	0.119	−0.169	0.165	−0.571	−0.011	−0.333	0.270
BA 19, L	−0.090	−0.032	0.126	−0.568	0.557	−0.173	−0.129
BA 4, L	−0.018	−0.241	−0.400	−0.515	0.145	0.344	0.033
BA 4, R	−0.026	−0.300	−0.371	−0.465	−0.192	0.248	0.132
BA 6, R	0.364	−0.313	−0.420	−0.424	−0.179	−0.059	0.088
BA 21, L	0.035	0.014	−0.110	0.009	0.862	0.041	0.129
BA 22, L	0.068	−0.179	0.051	−0.097	0.738	0.305	0.142
BA 37, L	−0.360	0.138	0.014	−0.156	0.719	−0.143	0.025
BA 20, L	−0.216	0.428	−0.139	0.145	0.718	−0.075	0.062
BA 40, L	0.196	−0.161	−0.034	−0.049	0.670	−0.337	−0.076
BA 39, L	−0.051	0.003	0.088	0.076	0.611	−0.541	−0.155
BA 13, R	0.075	0.198	0.151	0.394	−0.443	0.228	0.257
BA 39, R	0.135	−0.104	0.168	0.194	0.122	−0.728	0.316
BA 24, L	−0.005	0.077	0.512	0.372	−0.088	0.602	−0.063
BA 32, L	0.287	0.238	0.138	0.324	−0.075	0.599	−0.098
BA 32, R	0.286	0.254	0.304	0.276	−0.138	0.580	0.120
BA 24, R	0.041	0.138	0.550	0.275	−0.128	0.575	0.097
BA 40, R	0.318	−0.166	0.024	0.270	0.034	−0.536	0.236
BA 13, L	0.028	0.273	0.066	0.275	0.090	0.520	−0.162
BA 21, R	0.255	−0.047	−0.056	0.198	0.185	−0.200	0.789
BA 20, R	−0.097	0.327	−0.130	0.263	−0.026	−0.194	0.743
BA 36, R	−0.124	0.516	0.060	−0.061	−0.018	0.052	0.719
BA 37, R	−0.170	0.011	0.021	−0.170	−0.023	−0.392	0.698
BA 22, R	0.349	−0.224	0.147	−0.042	0.097	0.066	0.635
BA 38, R	0.016	0.428	−0.137	0.314	0.017	0.179	0.577
Eigenvalue	9.89	9.13	6.59	6.36	5.45	4.99	4.86
Percentage of variance^a^	14.55	13.43	9.70	9.35	8.01	7.34	7.15

*BA, Brodmann area; R, right; L, left.*

a *After varimax rotation*.

**Table 3:** Means and standard deviations of the factor scales in patients with MCI or AD and the healthy controls.

	**Factors**						
	**Frontal** ** cortex**	**Mesial temporal** ** substructures**	**Posterior** ** cingulum**	**Occipital** ** cortex**	**Left temporo-prefrontal** ** areas**	**Anterior** ** cingulum**	**Right temporal** ** cortex**
**AD**							
Mean	0.74	0.84	1.10	−1.21	1.02	0.43	1.01
SD	0.06	0.05	0.09	0.05	0.06	0.05	0.05
**MCI**							
Mean	0.75	0.86	1.12	−1.19	1.05	0.43	1.01
SD	0.05	0.06	0.08	0.07	0.05	0.05	0.05
**HC**							
Mean	0.79	0.99	1.05	−1.08	1.00	0.39	0.99
SD	0.044	0.07	0.07	0.07	0.07	0.07	0.04
Univariate *p*	0.007	0.000	0.014	0.000	0.000	0.124	0.281
Group comparisons	HC > MCI and AD	HC > MCI and AD	MCI > HC	HC > MCI and AD	MCI > HC and AD		

*Frontal cortex: F_(2, 126)_ = 5.15; mesial temporal substructures = F_(2, 126)_ = 27.57; posterior cingulum: F_(2, 126)_ = 4.44; occipital cortex: F_(2, 126)_ = 32.01; left temporo-prefrontal areas: F_(2, 126)_ = 8.45; anterior cingulum: F_(2, 126)_ = 2.13; right temporal cortex: F_(2, 126)_ = 1.28. AD, Alzheimer's disease; MCI, mild cognitive impairment; HC, healthy control; SD, standard deviation*.

## Discussion

The present study yielded three major findings: (i) support for the hypothesis that changes in the networks of cerebral sites, including the medial and ventrolateral prefrontal cortices, medial and lateral temporal cortices, temporoparietal junction, retrosplenial/posterior cortices, and cerebellar structures, contribute to decline in MCI and early AD; (ii) evidence that these changes may also involve compensatory efforts in patients with MCI; and (iii) an indication that both semantic and episodic AM deficits refer to changes in the mesial temporal substructures.

Cortical activity obtained in a resting state was segregated into seven dimensions of cortical activation, with lower values on the factors frontal cortex, mesial temporal substructures, and occipital cortex, differentiating patients with MCI and early AD patients from cognitively intact control subjects. This applies to all of the three factors mentioned above, although changes of the occipital cortices were less frequently reported (9). According to Svoboda et al. (7), the frontal cortices play an important role in conscious experience of re-experiencing, memory reconstruction, and self-referential processing. Mesial temporal substructures, in particular the hippocampi, are important for the encoding and retrieval of “laboratory” episodic memories (27), while the occipital cortex is important for visuospatial processing and visual imagery, but seems to play a merely marginal role for AM retrieval (7). However, significant group differences also involved increased values on the factors posterior cingulum and left temporo-prefrontal areas in patients with MCI when contrasted with controls and both AD patients and controls, respectively. A decreased glucose uptake is generally considered as an early and robust indicator of functional deficits and atrophy processes in the respective brain areas. In contrast, an increased value may indicate compensatory efforts due to an unspecific and less efficient overactivation, which was observed in both FDG-PET and fMRI studies. Further evidence supporting this hypothesis comes from studies in healthy subjects that yielded an economization of cerebral activation under training of cognitive tasks (28, 29). As emphasized by Matura et al. (27), the medial prefrontal cortex and the posterior cingulate cortex support self-referential processing as a defining component of AM; hence, most neuroimaging studies on AM yielded an activation during AM retrieval. One may hypothesize that the patients investigated in the present study were still capable of recalling semantic and episodic facts due to an overactivation of the respective areas. An activation of the occipital cortices was reported in studies contrasting recent relative to remote autobiographical events (7). Along with this, a decreased glucose uptake was reported in a number of studies, including our own trial, with significantly higher values in “good performers” of a serial verbal learning task among both patients and controls.

Besides this, both semantic and episodic AM deficits were significantly correlated with changes in the mesial temporal substructures, but not to other factors. The role of the hippocampi, gyri parahippocampi, and the perirhinal and entorhinal cortices as core contributors to AM was emphasized by Svoboda et al. (7); atrophic changes of the respective structure are among the best-established findings in AD (30, 31). That both domains of AM were highly significantly associated with the mesial temporal substructures conforms with the view that both are highly interrelated and do not constitute independent forms of AM. This finding indicates a fundamental, at least gradual, overlap between SAM and EAM [see, for example, (32)] and confirms the hypothesis that EAM is integrated into a higher-level external framework of these general knowledge structures (33).

While we included data from 129 patients who were subsequently recruited in a large memory clinic, AM was only obtained from 22 patients. The sample size of 22 subjects in the AM analysis increased the risk of type II errors. However, the core significant findings of our study are consistent with those in the review of Svoboda et al. (7), indicating that our sample size was sufficient for large effects. In their review, Svoboda and her co-workers (7) considered findings from both fMRI and PET studies. Systematic effects of the imaging method used were not reported. From a clinical standpoint, FDG-PET is well-established in the diagnostic workup of MCI and mild AD as it provides greater sensitivity in the early recognition of the cerebral changes in MCI and mild AD (34, 35) since functional deficits may precede morphological changes but already involve decreased glucose metabolic rates. FDG-PET provides a measure of network indicating which structures are correlated within subjects across the group. With PET, we demonstrate that parts of the frontal cortex are more similar to each other in functional activity in healthy subjects than in patients, and this is the concept of the network evaluated. With fMRI, additional measures of within-subject correlations over time can be demonstrated, offering an alternative but not entirely dissimilar assessment of a network. Taken together, our preliminary findings indicate that a similar network of cerebral sites important for AM in otherwise healthy and young controls also applies to patients with MCI and mild AD. Changes within this network include both activity decrease and activity increase, which may refer to compensatory efforts. Both episodic and semantic AM were significantly correlated with changes in the mesial temporal substructures. This confirms the hypothesis (33) that personal semantic knowledge constitutes a framework for episodic AM; hence, both referred to the same structure.

## Data Availability Statement

The raw data supporting the conclusions of this article will be made available by the authors, without undue reservation.

## Ethics Statement

The studies involving human participants were reviewed and approved by the ethics committee of the medical faculty of Heidelberg University. The patients/participants provided their written informed consent to participate in this study.

## Author Contributions

CF, UH, MB, and JS: substantial contributions to the conception or design of the work or the acquisition and analysis or interpretation of data for the work. CF, JS, JP, CD, MB, and CH: drafting the work or revising it critically for important intellectual content. All authors provide approval for publication of the content and agree to be accountable for all aspects of the work in ensuring that questions related to the accuracy or integrity of any part of the work are appropriately investigated and resolved.

## Funding

This research was funded by the Deutsche Forschungsgemeinschaft (471/5-1).

## Conflict of Interest

The authors declare that the research was conducted in the absence of any commercial or financial relationships that could be construed as a potential conflict of interest.

## Publisher's Note

All claims expressed in this article are solely those of the authors and do not necessarily represent those of their affiliated organizations, or those of the publisher, the editors and the reviewers. Any product that may be evaluated in this article, or claim that may be made by its manufacturer, is not guaranteed or endorsed by the publisher.
